# 5-(1*H*-Tetra­zol-5-yl)-1*H*-indole monohydrate

**DOI:** 10.1107/S1600536811018575

**Published:** 2011-05-28

**Authors:** Xing-Wei Cai, Hong-Fei Lu, Zhen Zhou

**Affiliations:** a School of Biological and Chemical Engineering, Jiangsu University of Science and Technology, Zhenjiang, Jiangsu 212003, People’s Republic of China; b Jiangsu University Environment Engineering, Inc., Zhenjiang, Jiangsu 212003, People’s Republic of China

## Abstract

In the title compound, C_9_H_7_N_5_·H_2_O, the inter­planar angles between the benzene and tetra­zole rings and between the benzene and imidazole rings are 8.71 (3) and 1.32 (2)°, respectively. In the crystal, strong N—H⋯N hydrogen bonds link the organic 5-(1*H*-tetra­zol-5-yl)-1*H*-indole mol­ecules into chains extended along the *b* axis. The chains are further inter­connected into layers parallel to (100) *via* strong O—H⋯N and N—H⋯O hydrogen bonds. Furthermore, the layers are inter­connected *via* strong O—H⋯N hydrogen bonds. Moreover, cohesion between the layers is provided by the π–π inter­actions between the imidazole, tetra­zole and benzene rings with centroid–centroid distances of 3.766 (2), 3.832 (2) and 3.733 (2) Å.

## Related literature

For applications of tetra­zole derivatives, see: Jin *et al.* (1994 ▶); Fu *et al.* (2009 ▶). For their use in the synthesis of metal-organic frameworks, see: Brewis *et al.* (2003 ▶). For related structures, see: Zhao *et al.* (2008 ▶); Fu *et al.* (2009 ▶). For the classification of hydrogen bonds, see: Desiraju & Steiner (1999 ▶).
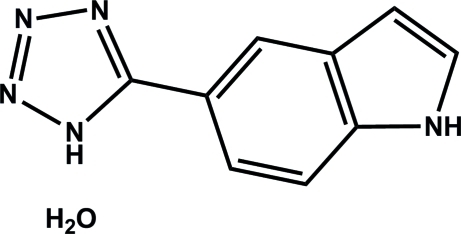


## Experimental

### 

#### Crystal data

C_9_H_7_N_5_·H_2_O
*M* *_r_* = 203.21
Orthorhombic, 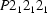

*a* = 6.8978 (14) Å
*b* = 9.953 (2) Å
*c* = 13.713 (3) Å
*V* = 941.4 (3) Å^3^
*Z* = 4
Mo *K*α radiation
μ = 0.10 mm^−1^
*T* = 298 K
0.40 × 0.30 × 0.20 mm

#### Data collection

Rigaku Mercury2 diffractometer
Absorption correction: multi-scan (*CrystalClear*; Rigaku, 2005 ▶) *T*_min_ = 0.89, *T*_max_ = 0.95
9741 measured reflections
1258 independent reflections
971 reflections with *I* > 2σ(*I*)
*R*_int_ = 0.069

#### Refinement

*R*[*F* ^2^ > 2σ(*F* ^2^)] = 0.045
*wR*(*F* ^2^) = 0.105
*S* = 1.06
1258 reflections
148 parameters
3 restraints
H atoms treated by a mixture of independent and constrained refinement
Δρ_max_ = 0.19 e Å^−3^
Δρ_min_ = −0.15 e Å^−3^

### 

Data collection: *CrystalClear* (Rigaku, 2005 ▶); cell refinement: *CrystalClear*; data reduction: *CrystalClear*; program(s) used to solve structure: *SHELXS97* (Sheldrick, 2008 ▶); program(s) used to refine structure: *SHELXL97* (Sheldrick, 2008 ▶); molecular graphics: *SHELXTL* (Sheldrick, 2008 ▶); software used to prepare material for publication: *SHELXTL*.

## Supplementary Material

Crystal structure: contains datablocks I, global. DOI: 10.1107/S1600536811018575/fb2235sup1.cif

Structure factors: contains datablocks I. DOI: 10.1107/S1600536811018575/fb2235Isup2.hkl

Supplementary material file. DOI: 10.1107/S1600536811018575/fb2235Isup3.cml

Additional supplementary materials:  crystallographic information; 3D view; checkCIF report

## Figures and Tables

**Table 1:** Hydrogen-bond geometry (Å, °)

*D*—H⋯*A*	*D*—H	H⋯*A*	*D*⋯*A*	*D*—H⋯*A*
N1—H1⋯N3^i^	0.87 (3)	2.18 (3)	3.042 (3)	171 (3)
O1*W*—H1*WA*⋯N2^ii^	0.80 (2)	2.08 (2)	2.869 (3)	173 (3)
O1*W*—H1*WB*⋯N4^iii^	0.79 (2)	2.33 (2)	3.098 (3)	164 (3)
N5—H5⋯O1*W*	0.91 (3)	1.85 (3)	2.757 (3)	173 (3)

Symmetry codes: (i) 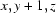; (ii) 
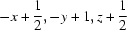
; (iii) 
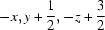
.

**Fig. 1.**
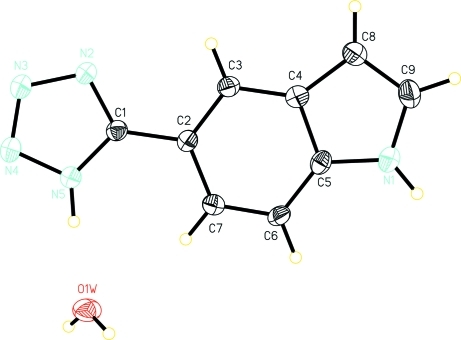
A view of the title molecules with the atomic numbering scheme. The displacement ellipsoids were drawn at the 30% probability level.

**Fig. 2.**
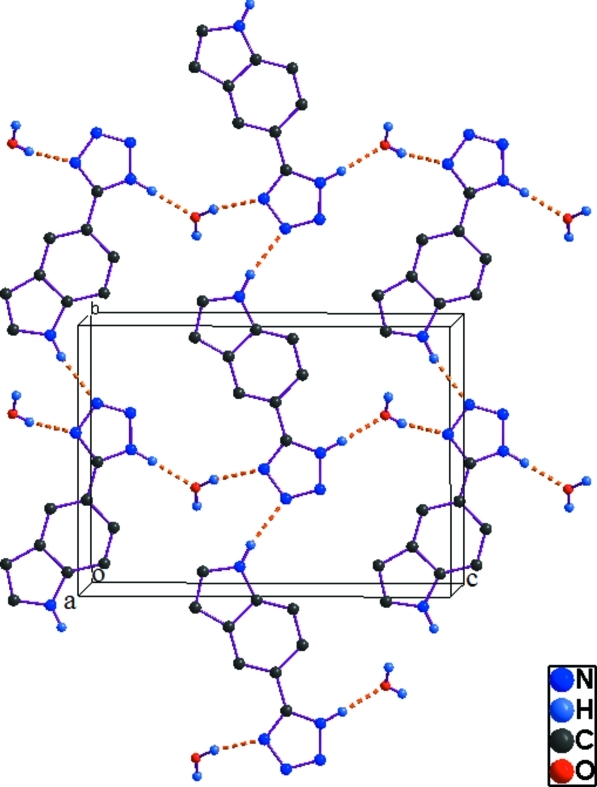
One layer with the atoms with the fractional coordinates of the constituting atoms x < 0.5. The H atoms not involved in the hydrogen bonding (dashed lines) have been omitted for clarity.

## supplementary crystallographic information

### Comment

The tetrazole functional derivatives have found a wide range of applications in coordination chemistry as ligands (Fu *et al.*, 2009), in medicinal chemistry as metabolically stable surrogates for the carboxylic acid group (Fu *et al.*, 2009) and in materials science as highly energetical materials for production of tetrazole explosives (Jin *et al.*, 1994). For the varying ability of the tetrazoles to coordinate metal ions, a large number of novel metal-organic frameworks have been synthesized (Brewis *et al.*, 2003). As extension of these works, we report here the crystal structure of the title compound, 5-(1*H*-tetrazol-5-yl)-1*H*-indole hydrate.

The interplanar angles between the benzene and the tetrazole and the benzene and imidazole rings equal to 8.71 (3)° and 1.32 (2)°, respectively. The geometric parameters of the tetrazole ring are comparable to those in the related molecules (Zhao *et al.*, 2008; Fu *et al.*, 2009).

The molecular packing is stabilized by strong intermolecular N—H···O, N—H···N and O—H···N hydrogen bonds (the classification of the hydrogen bonds is according to Desiraju & Steiner, 1999). The H-bonds link the molecules into a three-dimensional network (Fig. 2 and Tab. 1). In a more detail, N1-H1···N3 connects the molecules into chains extended along the *b*-axis. These chains are interconnected by the hydrogen bonds O1W—H1WA···N2 and N5—H5···O1W, forming thus layers parallel to (1 0 0) - see Fig. 2 and Tab. 1. The hydrogen bond O1W—H1WB···N4 interconnects the layers.

The layers are also connected by the π-electron ring··· π-electron ring interactions. The centroid—centroid distances are *Cg*1—*Cg*2 (1 + *x*, *y*, *z*) = 3.766 (2) Å; *Cg*1—*Cg*2 (1/2 + *x*, 3/2 - *y*, 1 - *z*) = 3.832 (2)Å and *Cg*3—*Cg*3 (1/2 + *x*, 3/2 - *y* or -1/2 + *x*, 3/2 - *y*, 1 - *z*) = 3.733 (2) Å , where *Cg*1, *Cg*2 and *Cg*3 are the centroids referring to the imidazole, tetrazole and the benzene rings, respectively.

### Experimental

5-(1*H*-tetrazol-5-yl)-1*H*-indole was obtained commercially from Alfa Aesar. Colourless block-shaped crystals suitable for X-ray analysis were obtained by slow evaporation of an ethanol/water (2:1 *v*/*v*) solution.

### Refinement

All the H atoms were discernible in the difference electron density maps. All the H atoms attached to the C atoms were situated into the idealized positions and treated as riding with C–H = 0.93 Å with *U_iso_*(H)=1.2*U_eq_*(C). The positional parameters of the H atoms involved in the hydrogen bonds were refined either freely (N1, N5) or as restrained (Ow). The restraints regarded the distances between the water oxygens and the water hydrogens (0.82 (2)Å) while the H1WA—O1w—H1WB angle was set to 105(1.5)°. The constraints regarding the displacement parameters of the amine and water hydrogens: *U_iso_*(H)=1.2*U_eq_*(N); *U_iso_*(H)=1.5*U_eq_*(Ow). Since there has been no significant anomalous scatterer in the structure, 889 Friedel pairs have been merged.

### Crystal data

**Table:**

C_9_H_7_N_5_·H_2_O	*F*(000) = 424
*M**_r_* = 203.21	*D*_x_ = 1.434 Mg m^−^^3^
Orthorhombic, *P*2_1_2_1_2_1_	Mo *K*α radiation, λ = 0.71073 Å
Hall symbol: P 2ac 2ab	Cell parameters from 1258 reflections
*a* = 6.8978 (14) Å	θ = 3.3–27.5°
*b* = 9.953 (2) Å	µ = 0.10 mm^−^^1^
*c* = 13.713 (3) Å	*T* = 298 K
*V* = 941.4 (3) Å^3^	Block, colourless
*Z* = 4	0.40 × 0.30 × 0.20 mm

### Data collection

**Table:**

Rigaku Mercury2 diffractometer	1258 independent reflections
Radiation source: fine-focus sealed tube	971 reflections with *I* > 2σ(*I*)
graphite	*R*_int_ = 0.069
Detector resolution: 13.6612 pixels mm^-1^	θ_max_ = 27.5°, θ_min_ = 3.3°
φ scans	*h* = −8→8
Absorption correction: multi-scan (*CrystalClear*; Rigaku, 2005)	*k* = −12→12
*T*_min_ = 0.89, *T*_max_ = 0.95	*l* = −17→17
9741 measured reflections	

### Refinement

**Table:**

Refinement on *F*^2^	Primary atom site location: structure-invariant direct methods
Least-squares matrix: full	Secondary atom site location: difference Fourier map
*R*[*F*^2^ > 2σ(*F*^2^)] = 0.045	Hydrogen site location: difference Fourier map
*wR*(*F*^2^) = 0.105	H atoms treated by a mixture of independent and constrained refinement
*S* = 1.06	*w* = 1/[σ^2^(*F*_o_^2^) + (0.0556*P*)^2^ + 0.0295*P*] where *P* = (*F*_o_^2^ + 2*F*_c_^2^)/3
1258 reflections	(Δ/σ)_max_ < 0.001
148 parameters	Δρ_max_ = 0.19 e Å^−^^3^
3 restraints	Δρ_min_ = −0.15 e Å^−^^3^
24 constraints	

### Special details

**Table:**

Experimental. The distance between the sample and the detector is 55 mm.
Geometry. All e.s.d.'s (except the e.s.d. in the dihedral angle between two l.s. planes) are estimated using the full covariance matrix. The cell e.s.d.'s are taken into account individually in the estimation of e.s.d.'s in distances, angles and torsion angles; correlations between e.s.d.'s in cell parameters are only used when they are defined by crystal symmetry. An approximate (isotropic) treatment of cell e.s.d.'s is used for estimating e.s.d.'s involving l.s. planes.
Refinement. Refinement of *F*^2^ against ALL reflections. The weighted *R*-factor *wR* and goodness of fit *S* are based on *F*^2^, conventional *R*-factors *R* are based on *F*, with *F* set to zero for negative *F*^2^. The threshold expression of *F*^2^ > σ(*F*^2^) is used only for calculating *R*-factors(gt) *etc*. and is not relevant to the choice of reflections for refinement. *R*-factors based on *F*^2^ are statistically about twice as large as those based on *F*, and *R*- factors based on ALL data will be even larger.

### Fractional atomic coordinates and isotropic or equivalent isotropic displacement parameters (Å^2^)

**Table:**

	*x*	*y*	*z*	*U*_iso_*/*U*_eq_	
N1	0.1423 (4)	1.0672 (2)	0.40317 (17)	0.0433 (6)	
H1	0.144 (5)	1.145 (3)	0.433 (2)	0.052*	
N5	0.1331 (4)	0.4891 (2)	0.61965 (15)	0.0371 (5)	
H5	0.120 (4)	0.532 (3)	0.6781 (19)	0.045*	
C2	0.1601 (4)	0.6777 (2)	0.49991 (17)	0.0296 (6)	
C3	0.1565 (4)	0.7101 (2)	0.40154 (17)	0.0339 (6)	
H3	0.1578	0.6428	0.3545	0.041*	
C7	0.1607 (4)	0.7798 (3)	0.57174 (18)	0.0357 (6)	
H7	0.1637	0.7561	0.6373	0.043*	
N4	0.1313 (4)	0.3538 (2)	0.61809 (16)	0.0456 (6)	
C5	0.1512 (4)	0.9450 (2)	0.44822 (18)	0.0339 (6)	
N3	0.1556 (4)	0.3203 (2)	0.52735 (17)	0.0471 (6)	
C8	0.1406 (5)	0.9156 (3)	0.28329 (19)	0.0441 (7)	
H8	0.1375	0.8770	0.2215	0.053*	
C1	0.1569 (4)	0.5364 (2)	0.52883 (18)	0.0308 (6)	
C4	0.1510 (4)	0.8459 (2)	0.37392 (18)	0.0334 (6)	
C6	0.1569 (4)	0.9126 (2)	0.54653 (18)	0.0362 (6)	
H6	0.1582	0.9794	0.5940	0.043*	
C9	0.1359 (5)	1.0484 (3)	0.3040 (2)	0.0471 (7)	
H9	0.1294	1.1169	0.2579	0.056*	
N2	0.1706 (4)	0.4307 (2)	0.47002 (16)	0.0421 (6)	
O1W	0.1242 (4)	0.6288 (2)	0.79322 (13)	0.0541 (6)	
H1WA	0.183 (4)	0.606 (3)	0.8404 (19)	0.081*	
H1WB	0.045 (4)	0.683 (3)	0.806 (2)	0.081*	

### Atomic displacement parameters (Å^2^)

**Table:**

	*U*^11^	*U*^22^	*U*^33^	*U*^12^	*U*^13^	*U*^23^
N1	0.0523 (14)	0.0299 (11)	0.0477 (14)	−0.0015 (15)	0.0014 (13)	−0.0001 (10)
N5	0.0484 (14)	0.0301 (11)	0.0328 (12)	−0.0004 (11)	0.0015 (13)	−0.0005 (10)
C2	0.0271 (13)	0.0275 (12)	0.0344 (13)	−0.0007 (13)	0.0031 (13)	−0.0015 (9)
C3	0.0377 (14)	0.0327 (13)	0.0312 (13)	−0.0018 (14)	−0.0009 (13)	−0.0034 (11)
C7	0.0409 (15)	0.0368 (14)	0.0292 (12)	−0.0006 (15)	0.0005 (13)	−0.0012 (11)
N4	0.0628 (15)	0.0275 (12)	0.0466 (14)	0.0006 (13)	0.0021 (15)	0.0031 (10)
C5	0.0290 (12)	0.0279 (12)	0.0447 (15)	0.0000 (14)	0.0015 (12)	−0.0013 (11)
N3	0.0662 (16)	0.0301 (12)	0.0450 (14)	−0.0044 (14)	0.0075 (15)	−0.0014 (10)
C8	0.0552 (18)	0.0421 (17)	0.0349 (15)	−0.0046 (16)	−0.0009 (15)	0.0029 (12)
C1	0.0299 (13)	0.0295 (13)	0.0331 (13)	−0.0031 (13)	0.0006 (13)	−0.0035 (11)
C4	0.0289 (12)	0.0355 (14)	0.0359 (14)	−0.0013 (13)	0.0009 (14)	−0.0039 (11)
C6	0.0449 (15)	0.0298 (14)	0.0338 (14)	0.0019 (14)	−0.0015 (14)	−0.0079 (10)
C9	0.0540 (18)	0.0397 (15)	0.0475 (17)	0.0002 (18)	0.0029 (16)	0.0124 (13)
N2	0.0601 (16)	0.0291 (11)	0.0370 (12)	−0.0023 (13)	0.0048 (12)	−0.0025 (10)
O1W	0.0737 (17)	0.0559 (14)	0.0327 (10)	0.0169 (13)	−0.0053 (11)	−0.0024 (10)

### Geometric parameters (Å, °)

**Table:**

N1—C5	1.366 (3)	N4—N3	1.299 (3)
N1—C9	1.373 (3)	C5—C6	1.387 (4)
N1—H1	0.87 (3)	C5—C4	1.418 (3)
N5—C1	1.341 (3)	N3—N2	1.354 (3)
N5—N4	1.347 (3)	C8—C9	1.353 (4)
N5—H5	0.91 (3)	C8—C4	1.425 (4)
C2—C3	1.387 (3)	C8—H8	0.9300
C2—C7	1.415 (3)	C1—N2	1.329 (3)
C2—C1	1.462 (3)	C6—H6	0.9300
C3—C4	1.403 (3)	C9—H9	0.9300
C3—H3	0.9300	O1W—H1WA	0.797 (17)
C7—C6	1.367 (3)	O1W—H1WB	0.792 (17)
C7—H7	0.9300		
			
C5—N1—C9	109.1 (2)	N4—N3—N2	111.0 (2)
C5—N1—H1	125.4 (19)	C9—C8—C4	107.1 (2)
C9—N1—H1	125.5 (19)	C9—C8—H8	126.5
C1—N5—N4	109.7 (2)	C4—C8—H8	126.5
C1—N5—H5	131.5 (18)	N2—C1—N5	107.1 (2)
N4—N5—H5	118.9 (18)	N2—C1—C2	126.6 (2)
C3—C2—C7	120.7 (2)	N5—C1—C2	126.2 (2)
C3—C2—C1	119.2 (2)	C3—C4—C5	118.4 (2)
C7—C2—C1	120.1 (2)	C3—C4—C8	134.8 (2)
C2—C3—C4	119.1 (2)	C5—C4—C8	106.7 (2)
C2—C3—H3	120.4	C7—C6—C5	118.1 (2)
C4—C3—H3	120.4	C7—C6—H6	120.9
C6—C7—C2	121.2 (2)	C5—C6—H6	120.9
C6—C7—H7	119.4	C8—C9—N1	109.9 (2)
C2—C7—H7	119.4	C8—C9—H9	125.1
N3—N4—N5	105.7 (2)	N1—C9—H9	125.1
N1—C5—C6	130.4 (2)	C1—N2—N3	106.5 (2)
N1—C5—C4	107.1 (2)	H1WA—O1W—H1WB	111 (2)
C6—C5—C4	122.5 (2)		

### Hydrogen-bond geometry (Å, °)

**Table:**

*D*—H···*A*	*D*—H	H···*A*	*D*···*A*	*D*—H···*A*
N1—H1···N3^i^	0.87 (3)	2.18 (3)	3.042 (3)	171 (3)
O1W—H1WA···N2^ii^	0.80 (2)	2.08 (2)	2.869 (3)	173 (3)
O1W—H1WB···N4^iii^	0.79 (2)	2.33 (2)	3.098 (3)	164 (3)
N5—H5···O1W	0.91 (3)	1.85 (3)	2.757 (3)	173 (3)

Symmetry codes: (i) *x*, *y*+1, *z*; (ii) −*x*+1/2, −*y*+1, *z*+1/2; (iii) −*x*, *y*+1/2, −*z*+3/2.
